# Increased Output of Recent Thymic Emigrants Contributes to Reconstitution of T‐Cell Pool During Recovery From COVID‐19

**DOI:** 10.1002/eji.70303

**Published:** 2026-10-29

**Authors:** Xiaobo Huang, Suvi T. Jokiranta, Kirsten Nowlan, Ngoc Anh Nguyen, Nelli Heikkilä, Lauriina Tola, Anu Kantele, Pirkka T. Pekkarinen, Johanna Hästbacka, Olli Vapalahti, Santtu Heinonen, Juuso Paajanen, Tomas Strandin, Eliisa Kekäläinen

**Affiliations:** ^1^ Translational Immunology Research Program University of Helsinki and Helsinki University Hospital Helsinki Finland; ^2^ Department of Bacteriology and Immunology University of Helsinki Helsinki Finland; ^3^ Human Microbiome Research Program Faculty of Medicine University of Helsinki Helsinki Finland; ^4^ Division of Intensive Care Medicine Department of Anaesthesiology and Intensive Care University of Helsinki and Helsinki University Hospital Helsinki Finland; ^5^ Faculty of Medicine and Health Technology Tampere University Hospital Wellbeing Services County of Pirkanmaa and Tampere University Tampere Finland; ^6^ Department of Virology, Medicum Viral Zoonoses Research Unit University of Helsinki Helsinki Finland; ^7^ HUSLAB Clinical Microbiology Division of Virology and Immunology HUS Diagnostic Center Helsinki Finland; ^8^ Department of Veterinary Biosciences University of Helsinki Helsinki Finland; ^9^ New Children's Hospital Pediatric Research Center University of Helsinki and Helsinki University Hospital Helsinki Finland; ^10^ Department of Pulmonology University of Helsinki and Helsinki University Hospital, Meilahti Hospital Helsinki Finland; ^11^ Helsinki Institute of Life Sciences HiLIFE University of Helsinki Helsinki Finland

**Keywords:** coronavirus disease 2019 (COVID‐19), homeostatic proliferation, naïve T cell, recent thymic emigrant

## Abstract

Acute coronavirus disease 2019 (COVID‐19) causes marked T‐cell lymphopenia, with recovery potentially driven by peripheral proliferation or increased thymic output of recent thymic emigrants (RTEs). We examined the relative contribution of these mechanisms by analyzing naïve T‐cell and RTE dynamics in 63 patients sampled at 1–3 timepoints during and after severe acute respiratory syndrome coronavirus 2 (SARS‐CoV‐2) infection. Overall frequencies of naïve CD4+ and CD8+ T cells remained stable during convalescence. In a female‐biased subcohort with paired samples, CD31+ CD4+ and CD21+ CD8+ RTE‐enriched subset frequencies increased from acute to late convalescence (LC) stage. Across the full cohort, a significant increase was confirmed for CD21+ CD8+ RTE‐enriched subsets. PD‐1 and Ki67 expression did not explain this rise, indicating enhancement of thymic output despite the cohort's high median age. Patients requiring ICU care had lower CD31+ CD4+ RTE‐enriched subset frequencies in LC compared with non‐ICU cases. Regression coefficient modeling identified male sex as a negative predictor of CD31+ CD4+ RTE‐enriched subset frequency, potentially contributing to distinct CD31+ CD4+ and CD21+ CD8+ RTE‐enriched subset kinetics. Together, these findings suggest that de novo thymic output, particularly CD21+ CD8+ RTE‐enriched subset, contributes to T‐cell reconstitution after COVID‐19 and may influence susceptibility to post‐infectious immune dysregulation.

## Graphical abstract

Acute COVID‐19 causes marked T‐cell lymphopenia, and how the pool recovers is unclear. Studying 63 patients across acute and convalescent phases, we found that naïve T‐cell frequencies stayed stable, whereas recent thymic emigrant (RTE)‐enriched subsets rose during recovery. Because this increase was independent of proliferation, it points to renewed thymic output driving T‐cell reconstitution, even in a cohort with a mean age of 55 years.

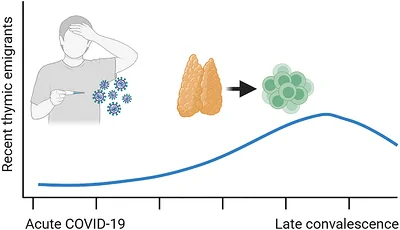

**Abbreviations**
COVID‐19: coronavirus disease 2019
dsso: days since symptom onset
EC: early convalescent
FDR: false discovery rate
LC: late convalescent
PBMC: peripheral blood mononuclear cell
RTEs: recent thymic emigrants
SARS‐CoV‐2: severe acute respiratory syndrome coronavirus 2
TRECs: T‐cell receptor excision circles
Tscm: stem cell‐like memory T cell

## 1 Introduction

Acute severe coronavirus disease 2019 (COVID‐19) is characterized by pronounced lymphopenia, which is mainly driven by a decline in circulating T cells, and is associated with adverse clinical outcomes [1]. This T‐cell depletion may result from direct viral‐mediated T‐cell destruction [1], activation‐induced lymphocyte redistribution [2], or acute infection‐associated thymic atrophy, which leads to reduced thymic output [3]. The magnitude of naïve T‐cell loss correlates with disease severity, suggesting preservation of this population is critical for controlling viral replication [4].

Recovery of the T‐cell pool after acute lymphopenia involves restoration of T‐cell frequencies through two mechanisms: peripheral proliferation of mature T cells and thymic output of newly generated T cells [5, 6]. Recent thymic emigrants (RTEs) are a T‐cell population consisting of cells recently egressed from the thymus and represent a specialized population essential for immune reconstitution [7]. Thymic dysfunction during severe COVID‐19 has been proposed to impair RTE output and prolong immunodeficiency, whereas enhanced RTE production during recovery could restore a diverse naïve T‐cell compartment [6]. The T‐cell pool can also recover through lymphopenia‐induced proliferation, which is considered a risk factor for developing autoimmunity because autoreactive clones gain a competitive advantage during the proliferation [8]. On the basis of these mechanisms, we decided to characterize RTE contributions to immune restoration after acute COVID‐19 to elucidate if the immune reconstitution could pose a risk for immune dysregulation after the acute infection.

## 2 Results and Discussion

### 2.1 Naïve T‐Cell Pool Stays Stable in COVID‐19

To evaluate the kinetics of naïve T cells throughout COVID‐19, we collected 90 prospective peripheral blood mononuclear cell (PBMC) samples from 63 individuals with PCR‐confirmed severe acute respiratory syndrome coronavirus 2 (SARS‐CoV‐2) infection.

The samples were categorized into acute (*n* = 24), early convalescent (EC) (*n* = 22), and late convalescent (LC) (*n* = 44) groups and analyzed using multicolor cytometry (Table S2) with gating strategy shown in Figure S6. The clinical characteristics of the patients are detailed in Table S1. Using a restricted maximum likelihood (REML) linear mixed‐effects model, we could detect no sustained increase or decrease of CD4+ and CD8+ naïve T cells when evaluated against time (days) since symptom onset (dsso) (Figure 1A,B). When we compared the frequency of CD4+ and CD8+ naïve T cells in a subcohort (*n* = 12, 67% females, median age 59 years) where paired samples were available from the acute and LC stages, no significant changes were observed (Figure 1C,D). These findings suggest that despite marked interindividual variation, the naïve T‐cell population remains stable throughout the course of COVID‐19. The observed significant correlation between age and naïve T‐cell frequencies in this study (Figure S1A,F) is consistent with the well‐established decline in naïve T‐cell frequencies during aging [9]. The preserved naïve T‐cell pool during and after COVID‐19 suggests that homeostatic maintenance of antigen‐inexperienced T cells remains largely intact, which is contrary to the well‐described lymphopenia affecting other T‐cell subsets, such as mucosal‐associated invariant T cells during acute disease [10].

**FIGURE 1:**
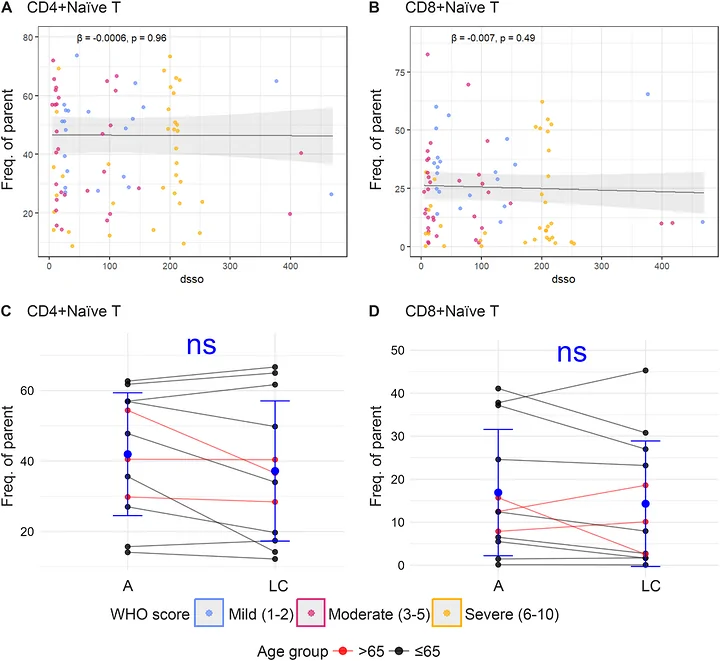
Naïve T‐cell frequencies remain stable throughout COVID‐19. (A and B) Longitudinal CD4+ and CD8+ naïve T‐cell kinetics across 63 patients stratified by dsso. (C and D) Paired analysis of matched samples (*n* = 12) comparing acute and late convalescent (LC) stages. Frequencies represent naïve T cells within total CD4+ or CD8+ T cells. Patients are color‐coded by WHO severity (mild = blue, moderate = red, and severe = yellow) and age (<65 years = black, ≥65 years = red). Linear regression coefficients (*β*) and *p* values are shown. Paired *t*‐test or Wilcoxon signed‐rank test with FDR correction was used. Data are presented as mean ± SD; ns = not significant.

### 2.2 Frequency of CD21+ CD8+ RTE‐Enriched Subset Is Increased During Recovery After Acute COVID‐19

Next, as a direct estimation of the cellular thymic output, we compared the frequency of RTEs in acute and LC COVID‐19 stages. In paired samples, CD31+ CD4+ RTE‐enriched subset frequency increased significantly from acute to LC stage (Figure 2A). This increase persisted after exclusion of CD95+ stem cell‐like memory T cells (Tscms), which can skew naïve T‐cell analysis if not excluded (Figure 2B) [11]. Furthermore, the increase was not attributable to recently activated PD‐1+ CD31+ CD4+ RTE‐enriched subset [12] (Figure 2C) or homeostatic proliferation, as measured by Ki67 expression (Figure 2D).

**FIGURE 2:**
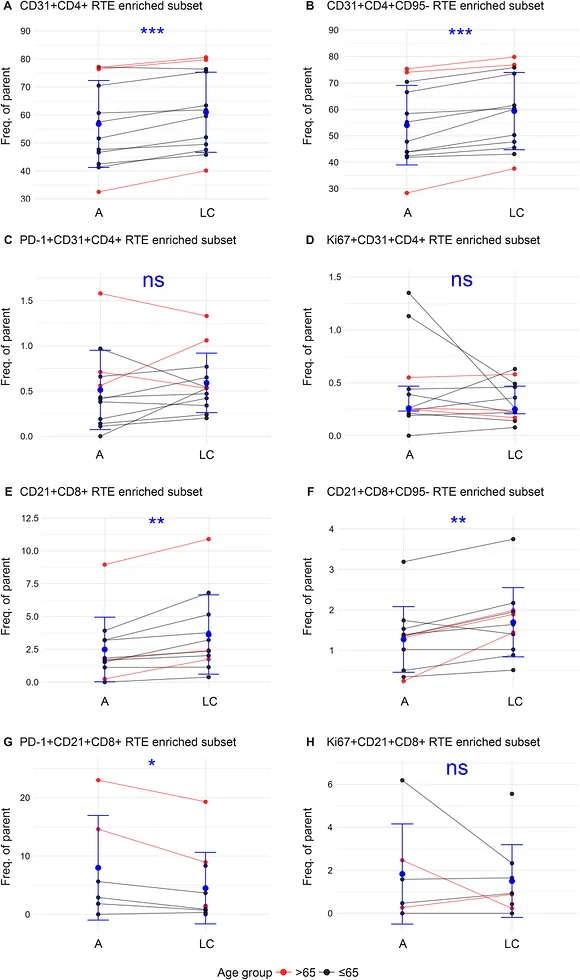
Lower CD31+ CD4+ and CD21+ CD8+ RTE‐enriched subset frequencies during acute COVID‐19 with concurrent elevation of PD‐1 expression on CD21+ CD8+ RTE‐enriched subsets. (A–H) Paired analysis of matched samples (*n* = 12) comparing acute and late convalescent (LC) stages. Panels show frequencies of CD31+ CD4+ RTE‐enriched subsets (A and B), PD‐1+ or Ki67+ CD31+ CD4+ RTE‐enriched subsets (C and D) within CD4+ naïve T cells, and CD31+ CD4+ RTE‐enriched subsets, respectively. CD21+ CD8+ RTE‐enriched subsets (E and F), PD‐1+ or Ki67+ CD21+ CD8+ RTE‐enriched subsets (G and H) within CD8+ naïve T cells and CD21+ CD8+ RTE‐enriched subsets, respectively. Patients >65 years are marked in red; ≤65 years in black. Paired *t*‐test or Wilcoxon signed‐rank test with FDR correction was applied. Data shown as mean ± SD (blue); *p < 0.05, **p < 0.01, ***p < 0.001; ns = not significant.

We next analyzed CD8+ RTE‐enriched subset and used CD21 as a marker to separate CD8+ RTE‐enriched subset from total naïve CD8+ T‐cell pool [13]. Similarly to CD31+ CD4+ RTE‐enriched subset, CD21+ CD8+ RTE‐enriched subset frequency increased significantly from acute to LC stage, and CD95+ Tscm exclusion did not affect this result (Figure 2E,F). A slight decrease in recently activated PD‐1+ CD21+ CD8+ RTE‐enriched subsets was observed from the acute stage (Figure 2G), whereas proliferating Ki67+ CD21+ CD8+ RTE‐enriched subset frequency remained stable.

We also examined effector memory (TEM), central memory (TCM), and terminally differentiated effector memory T‐cell (TEMRA) frequencies but found no significant changes between acute and LC disease stages (Figure S2A,E,I,M). The significantly higher frequencies of Ki67 and PD‐1 on CD4+ TEMs, TCMs, TEMRAs (Figure S2B,D,F,H,L), and CD8+ TEMRAs (Figure S2N,P) in acute versus LC stages again confirmed the antigen‐driven activation during the acute phase of COVID‐19, which resolves after viral clearance.

When we analyzed the same RTE populations from the whole cohort with REML, the CD31+ CD4+ and CD31+ CD4+ CD95− RTE‐enriched subset frequencies did not significantly change over time (Figure S3A,B). Instead, variation in CD31+ CD4+ and CD31+ CD4+ CD95− RTE‐enriched subset frequencies was better explained by other variables: Age, male sex, and disease severity, as measured with the WHO scoring system, had all inverse correlation with CD31+ CD4+ and CD31+ CD4+ CD95− RTE‐enriched subset frequencies (Figure S1B,C). PD‐1+ CD31+ CD4+ RTE‐enriched subsets increased over time (beta coefficient 0.0008 for dsso) (Figure S3C), but this effect was primarily driven by age rather than increased proliferation (Figure S1D, Figure 2D), consistent with recently reported age‐associated upregulation of baseline PD‐1 on naïve T cells [14].

On the contrary, the effect of increased CD21+ CD8+ RTE‐enriched subset frequency over time remained in the whole cohort, although the effect was modest (log beta‐coefficient 0.328, Figure S3E, log beta‐coefficient 0.216, Figure S3F). Unlike CD31+ CD4+ RTE‐enriched subsets, CD21+ CD8+ RTE‐enriched subsets were not as strongly confounded with age (Figure S1G,H). The frequencies of PD‐1 and Ki67‐positive CD21+ CD8+ RTE‐enriched subsets did not show significant variance over time (Figure S3G,H).

Overall, these results support the conclusion that RTE‐enriched subset frequencies increase during recovery from COVID‐19, with the paired‐sample findings providing the more sensitive estimate of this recovery‐phase trend. Renewed thymic output of CD21+ CD8+ RTE‐enriched subset likely contributes to correction of the CD8+ T‐cell lymphopenia after acute COVID‐19. Detected differences between the paired samples on CD31+ CD4+ (Figure S4A,B) and CD21+ CD8+ RTE‐enriched subset can be explained by the higher proportion of females in the paired samples (Figure S4C). The maintained thymic output in later life in females has been speculated [15].

### 2.3 Intensive Care Unit‐Treated COVID‐19 Patients Have a Lower Frequency of CD4+ RTE‐Enriched Subset During LC

We compared the frequencies of RTE‐enriched subsets in the LC phase from patients treated in the ICU with those who did not require ICU treatment during the acute phase. No significant differences between the groups were observed in terms of age or time of sampling (Table S3). Similar to the paired samples analysis, we did not observe any differences in naïve T‐cell frequencies between ICU‐ and non‐ICU‐treated patients (Figure 3A,D). We observed a significantly lower frequency of CD31+ CD4+ RTE‐enriched subset in individuals who were ICU‐treated, but the difference for CD21+ CD8+ RTE‐enriched subset was not statistically significant (Figure 3B,E). Lower frequencies of CD31+ CD4+ RTE‐enriched subsets in severe COVID‐19 suggest that thymic output of helper T cells is particularly compromised in patients with the highest inflammatory burden, which is in line with a previous report of lower T‐cell receptor excision circles (TREC), another widely used proxy for thymic output, in ICU‐treated COVID‐19 patients [6]. However, to date, studies measuring TREC in COVID‐19 patients have not differentiated between CD4+ and CD8+ RTEs. Thus, our flow cytometry–based analysis gives an increasingly refined picture of how thymic output contributes to the reconstitution of the T‐cell pool during COVID‐19.

**FIGURE 3:**
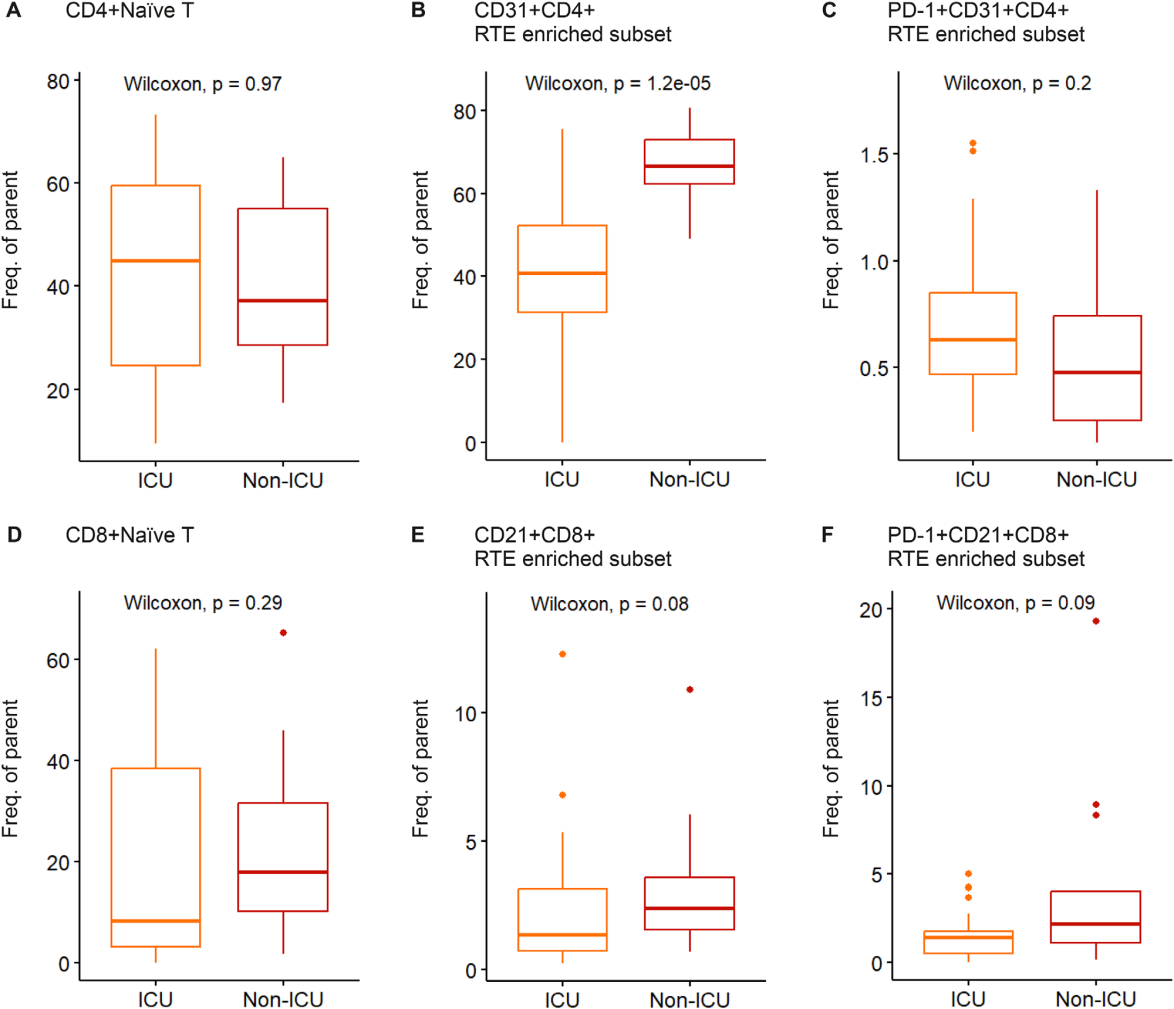
RTE‐enriched subset frequencies are reduced during recovery from severe COVID‐19 requiring intensive care. (A–F) Comparative analysis of naïve T cells and recent thymic emigrant populations in convalescent patients recovering from severe COVID‐19 requiring ICU treatment (*n* = 30) versus non‐ICU patients (*n* = 14). (A) The frequency of naïve T cells in all CD4+ T cells. (B) The frequency of CD31+ RTE‐enriched subsets in CD4+ naïve T cells. (C) The frequency of PD‐1+ CD31+ RTE‐enriched subsets in CD4+ naïve T cells. (D) The frequency of naïve T cells in CD8+ T cells. (E) The frequency of CD21+ CD8+ RTE‐enriched subsets in CD8+ naïve T cells. (F) The frequency of PD‐1+ CD21+ CD8+ RTE‐enriched subsets in CD8+ naïve T cells. Wilcoxon signed‐rank tests were used with comparison.

### 2.4 Plasma IL‐15 Concentration Is Increased During Acute COVID‐19

Finally, to explore potential mechanisms underlying the post‐infection increase in RTE‐enriched subsets, we measured plasma IL‐7 and IL‐15, which, respectively, support naïve and memory T‐cell homeostasis, survival, and proliferation [16]. We used paired acute and LC samples from 11 patients and additional unpaired LC samples from 7 patients. IL‐7 concentration did not differ between stages in either paired or unpaired analysis (Figure S5A,B), suggesting that the observed pattern is unlikely to be a passive consequence of lymphopenia‐driven proliferation [17]. In contrast, IL‐15 concentrations were significantly higher during acute stage in both analyses (Figure S5C,D). The decline in IL‐15 during LC coincided with increased CD21+ CD8+ RTE‐enriched subset frequencies. This pattern is consistent with previous findings linking higher IL‐15 to greater disease severity during acute COVID‐19 [16] and suggests that factors beyond circulating IL‐15 contribute to thymic output and T‐cell reconstitution.

## 3 Data Limitations and Perspectives

Our study is constrained by a relatively small cohort and subgroup size. Although no multicollinearity was detected in our multivariable analysis, the variables are known to depend on each other (such as age and disease severity); the results must be interpreted with caution. Convenience sampling resulted in an imbalanced sex distribution in paired samples, which means that our findings cannot be generalized and should be considered more exploratory. Furthermore, due to incomplete patient treatment data, we were unable to analyze the influence that potentially differing treatment regimens may have on thymic output during hospitalization.

RTE identification was limited by the absence of CD38 in our panel. Although CD38 is a robust RTE marker [18], its use in acute or highly inflammatory settings requires further validation because CD38 is also strongly upregulated during T‐cell activation, including acute SARS‐CoV‐2 infection [19]. We, therefore, relied on established surrogate markers that may not fully represent the complete RTE compartment. CD21 was used as an operational marker for CD8+ RTE‐enriched subset, but its expression may also be affected by inflammatory or cytokine‐driven peripheral activation and may not uniquely identify bona fide RTEs during acute or post‐viral recovery. Altogether, CD8+ RTE markers are less well established than CD31 for CD4+ RTEs [7].

Limited sample volume prevented TREC quantification, which could have provided additional evidence of thymic output. Furthermore, the absence of baseline RTE measurements prior to SARS‐CoV‐2 infection limits assessment of individual changes associated with COVID‐19. Larger, prospectively designed, and sex‐balanced cohorts with validated RTE markers and direct measures of thymic output are needed to clarify the relationships between disease severity, thymic output, and immune recovery.

## 4 Concluding Remarks

In conclusion, RTE‐enriched subset frequencies increased during recovery from COVID‐19, whereas ICU‐treated patients had lower CD4+ RTE‐enriched subset frequencies at the LC stage. The lower frequencies in ICU patients may reflect or contribute to severe disease. The observed increase in RTE‐enriched subset was notable given the cohort's mean age of 55 years, when thymic output is generally considered reduced. Our findings, together with evidence of protective thymic hyperplasia and functional thymic tissue in older individuals [20, 21], suggest that reactive thymic activity may contribute to T‐cell recovery beyond midlife. The association of female sex with higher CD4+ RTE‐enriched subset frequencies further supports a potential role for sex‐related differences in thymic function.

However, the mechanisms underlying these changes, particularly the increase in CD21+ CD8+ RTE‐enriched subsets, remain unclear. Larger studies incorporating validated RTE markers, cytokine profiling, thymic imaging, and sex‐balanced longitudinal cohorts are needed to distinguish increased thymic output from peripheral T‐cell proliferation and to determine their relevance to immune recovery and longer term immune dysregulation.

## Author Contributions

Conceptualization: Nelli Heikkilä, Tomas Strandin, and Eliisa Kekäläinen. Methodology: Xiaobo Huang and Nelli Heikkilä. Software: Xiaobo Huang and Suvi T. Jokiranta. Validation: Xiaobo Huang. Formal analysis: Xiaobo Huang and Suvi T. Jokiranta. Investigation: Xiaobo Huang, Suvi T. Jokiranta, Kirsten Nowlan, Ngoc Anh Nguyen, Lauriina Tola, and Santtu Heinonen. Resources: Anu Kantele, Pirkka T. Pekkarinen, Johanna Hästbacka, Olli Vapalahti, Tomas Strandin, and Eliisa Kekäläinen. Data curation: Xiaobo Huang and Juuso Paajanen. Visualization: Xiaobo Huang, Suvi T. Jokiranta, and Eliisa Kekäläinen. Supervision: Nelli Heikkilä, Tomas Strandin, and Eliisa Kekäläinen. Project administration and funding acquisition: Eliisa Kekäläinen. Writing – original draft: Xiaobo Huang, Suvi T. Jokiranta, and Eliisa Kekäläinen. All authors reviewed and approved the manuscript.

## Ethics Statement

The study was approved by the Ethics Committee of the Hospital District of Helsinki and Uusimaa (HUS/853/2020, HUS/1238/2020).

## Consent

All patients gave written informed consent in accordance with the Declaration of Helsinki.

## Conflicts of Interest

The authors declare no conflicts of interest.

## Supporting information




**Supporting File**: eji70303‐sup‐0001‐SuppMat.pdf.

## Data Availability

The data used during the current study are available from the corresponding author on reasonable request.
