# Supplementary material for: Increased Output of Recent Thymic Emigrants Contributes to Reconstitution of T‐Cell Pool During Recovery From COVID‐19
Source: Eur J Immunol. 2026 Oct 29;56(10):e70303. doi: 10.1002/eji.70303 (PMC13718309; doi:10.1002/eji.70303)
Supplement: Supplementary file 1 [file EJI-56-e70303-s001.pdf]

Huang X et al. Increased output of recent thymic emigrants contributes to reconstitution of T-cell pool during recovery from COVID-19

Supporting Information

Materials and methods

Patients

SARS-CoV-2-infected patients aged 27 to 78 years (n=63) with RT-PCR-confirmed acute or convalescent COVID-19 were recruited from Helsinki University Hospital, Finland. Blood samples were collected at various time points post-symptom onset and categorized as acute (<21 days, n=24), early convalescent (21–90 days, n=22), and late convalescent (>90 days, n=44). Demographic details are provided in Supporting Table 1. Disease severity was classified using the WHO Clinical Progression Scale (2020) (1), with scores 1–2 as mild, 3–5 as moderate, and 6–10 as severe.

Supporting Information Table 1. Clinical information of COVID-19 patients

**Characteristic**		**All patients**	**Acute sample group**	**Early convalescent sample group**	**Late convalescent sample group**	**Paired Acute&Late convalescent samples**
n (%)		63 (100)	24 (100)	22 (100)	44 (100)	12 (100)
Treatment condition (%)	ICU	33 (52)	8 (33)	3 (14)	30 (68.0)	7 (58.0)
	Non-ICU	30 (48)	16 (67)	19 (86)	14 (32.0)	5 (42.0)
Age, (median)	years	55.0	56.0	52.0	58.0	59.0

Age, years IQR	25th	44.0	40.0	34.0	49.0	56.0
	75th	64.0	66.0	63.0	65.0	66.0
Female (%)		31 (49.0)	14 (58.0)	14 (64.0)	21 (48.0)	8 (67.0)
Male (%)		32 (51.0)	10 (42.0)	8 (36.0)	23 (52.0)	4 (33.0)
Dsso (median)		-	11	28	199	-
Dsso range		-	2 - 20	21 - 89	91 - 467	-
WHO score Mild (1-2) (%)		-	0 (0)	13 (59.0)	8 (18.0)	-
WHO score Moderate (3-5) (%)		-	16 (67.0)	5 (23.0)	7 (16.0)	-
WHO score Severe (6-10) (%)		-	8 (33.0)	4 (18.0)	29 (66.0)	-

IQR: Interquartile range; Dsso: Days since symptom onset; 2 samples overlapped in all three groups; 12 samples overlapped in acute and late convalescent groups.

Cell Isolation and Flow Cytometry

PBMCs from COVID-19 patients and follow-up samples were isolated by Ficoll-Paque PLUS (Cytiva) density gradient centrifugation, cryopreserved using the CTL Freezing Kit (ImmunoSpot), and stored in liquid nitrogen until analysis. Before staining, PBMCs were rapidly thawed at 37°C, washed with CTL wash (ImmunoSpot), and stained with surface and

viability markers for 30 min at 4°C in Horizon Brilliant Staining Buffer (BD). The antibodies used for staining are listed in Supporting Table 2. We acknowledge that CD21+ is not a definitive marker of CD8+ thymic emigrants. In this study, the CD21+ CD8+ population is used as an operational proxy for an RTE-enriched subset, based on our prior experience with using it as a RTE marker (2). Live/dead discrimination was performed using the LIVE/DEAD Fixable Green (Thermo Fisher Scientific). Cells were then fixed and permeabilized using the FoxP3/Transcription Factor Fixation/Permeabilization Kit (eBioscience) for 40 min at 4°C, followed by intracellular staining in 1× Permeabilization Buffer (eBioscience) for 30 min at 4°C. Samples were acquired on a BD Fortessa LSRII flow cytometer (BD) and analyzed with FlowJo v10.10.0. The gating strategy is shown in Fig. S6.

Supporting Information Table 2. Antibodies used in each panel

Antigen	Fluorochrome	Clone	Host	Cat number	Manufacturer
Panel for T-cell kinetics during COVID-19 recovery					
CD19	FITC	HI19a	Mouse	21810083	Immunotools
CD14	FITC	18D11	Mouse	21620143	Immunotools
PD-1	PE	EH12.2H7	Mouse	329906	Biolegend
CCR7	PE-CF594	150503	Mouse	562381	BD
CD31	PE-Cy7	WM59	Mouse	563651	BD
CD8	APC-R700	RPA-T8	Mouse	565181	BD
CD3	APC-H7	SK7	Mouse	560176	BD
CD95	BV421	DX2	Mouse	562616	BD
CD4	BV510	SK3	Mouse	562970	BD

TCRgd	BV605	11F2	Mouse	745202	BD
CD21	BV650	B-ly4	Mouse	740569	BD
CD45RA	BV711	HI100	Mouse	563870	BD
Ki67	BV786	B56	Mouse	563756	BD
FoxP3	APC	236A/E7	Mouse	17-4777-42	eBioscience

Cytokine measurements

Plasma concentrations of IL-7 and IL-15 in acute and LC samples were measured using the Simple Plex Ella™ Automated ELISA platform (Bio-Techne, Minneapolis, MN, USA) according to the manufacturer's instructions. System performance was verified with a quality-control cartridge before sample processing. Samples and reagents were equilibrated to room temperature, and plasma samples were diluted 1:2. A volume of 50 µL of each diluted sample was loaded into the designated wells of the Ella cartridge. Similarly, 50 µL of the reconstituted lyophilized controls were added to the corresponding control wells. Wash buffer was added to the reservoir wells in accordance with the manufacturer's protocol. The assay was run using the Simple Plex Runner, and the resulting data were analyzed in R.

Statistical analysis

Statistical analyses were performed in R (v4.5.1). Linear temporal effects, accounting for inter-patient variability and confounding factors, were assessed using a REML linear mixed-effects model with the formula: variable ~ dsso + who_max + sex + age + (1 | patient). Only random intercepts were included, and dsso was log-transformed where appropriate. Modeling and visualization were performed using the lme4 (3) and sjPlot (4) packages, respectively. Paired t-tests or Wilcoxon signed-rank tests with False Discovery Rate (FDR) correction were applied for paired comparisons based on data distribution. Results are expressed as mean ± SD, and statistical significance was defined as $P<0.05$ (*$P<0.05$, **$P<0.01$, ***$P<0.001$; ns=not significant).

Supporting Information Table 3. Descriptive Characteristics of Participants in the ICU and Non-ICU Groups

Characteristic	Statistic	ICU	Non-ICU
Age, years	Count	30	14
	Mean	57.4	52.1
	Median	59	57.5
	Minimum	33	27
	Maximum	78	73
Dsso	Count	30	14
	Mean	185	208
	Median	206	141
	Minimum	99	91
	Maximum	254	467

Dsso: Days since symptom onset.

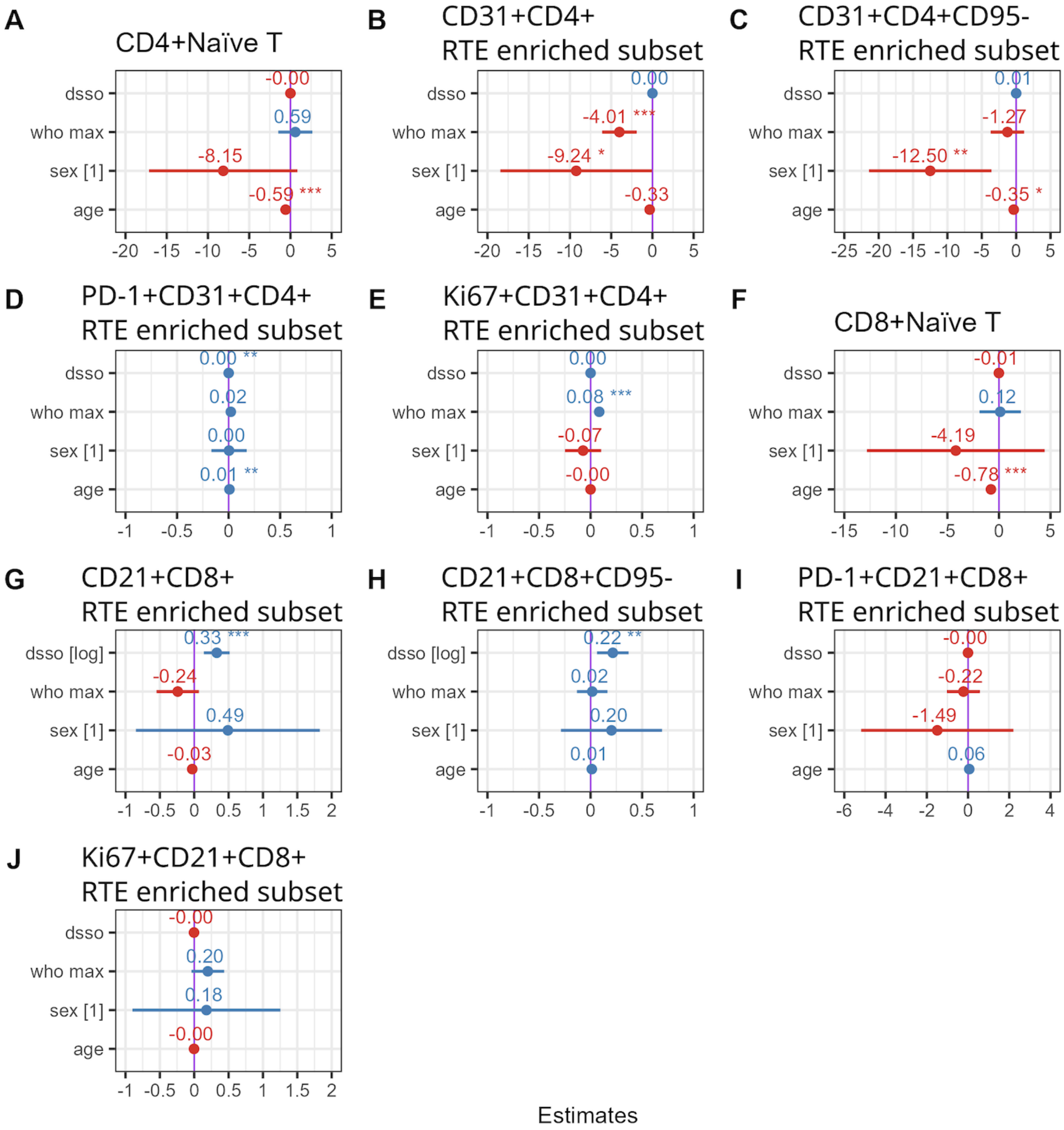

Supporting Information Figure 1. Effects associated with CD4+ and CD8+ Naïve T cells, RTE-enriched subset and their subpopulations in SARS-CoV-2 infection. Forest plot displaying standardized regression coefficient estimates (betas) and 95% confidence intervals from a mixed-effects model analyzing effects associated with (A) CD4+ naïve T cells. (B) CD31+ CD4+ RTE-enriched subset. (C) CD31+ CD4+ CD95- RTE-enriched subset. (D) PD-1+ CD31+ CD4+ RTE-enriched subset. (E) Ki67+ CD31+ CD4+ RTE-enriched subset. (F) CD8+ naïve T cells. (G) CD21+ CD8+ RTE-enriched subset. (H) CD21+ CD8+ CD95- RTE-enriched subset. (I) PD-1+ CD21+ CD8+ RTE-enriched subset. (J) Ki67+ CD21+ CD8+ RTE-enriched subset in peripheral blood of COVID-19 patients. The model examined four independent variables: days since symptom onset (dsso, continuous), WHO severity classification (who max 1-10, continuous), biological sex (sex where 1=male, binary), and age

(years, continuous). Each estimate indicates the percent change in the T-cell subset frequency per one-unit increase in the predictor variables. Estimates are presented on the x-axis; estimates crossing the vertical reference line at zero indicate no significant association with the cell frequency. The horizontal lines of each effect represent the 95% confidence interval. Linear regression coefficients (β) are shown. Red points indicate negative associations; blue points indicate positive association. *$P<0.05$, *$P<0.01$, ***$P<0.001$.

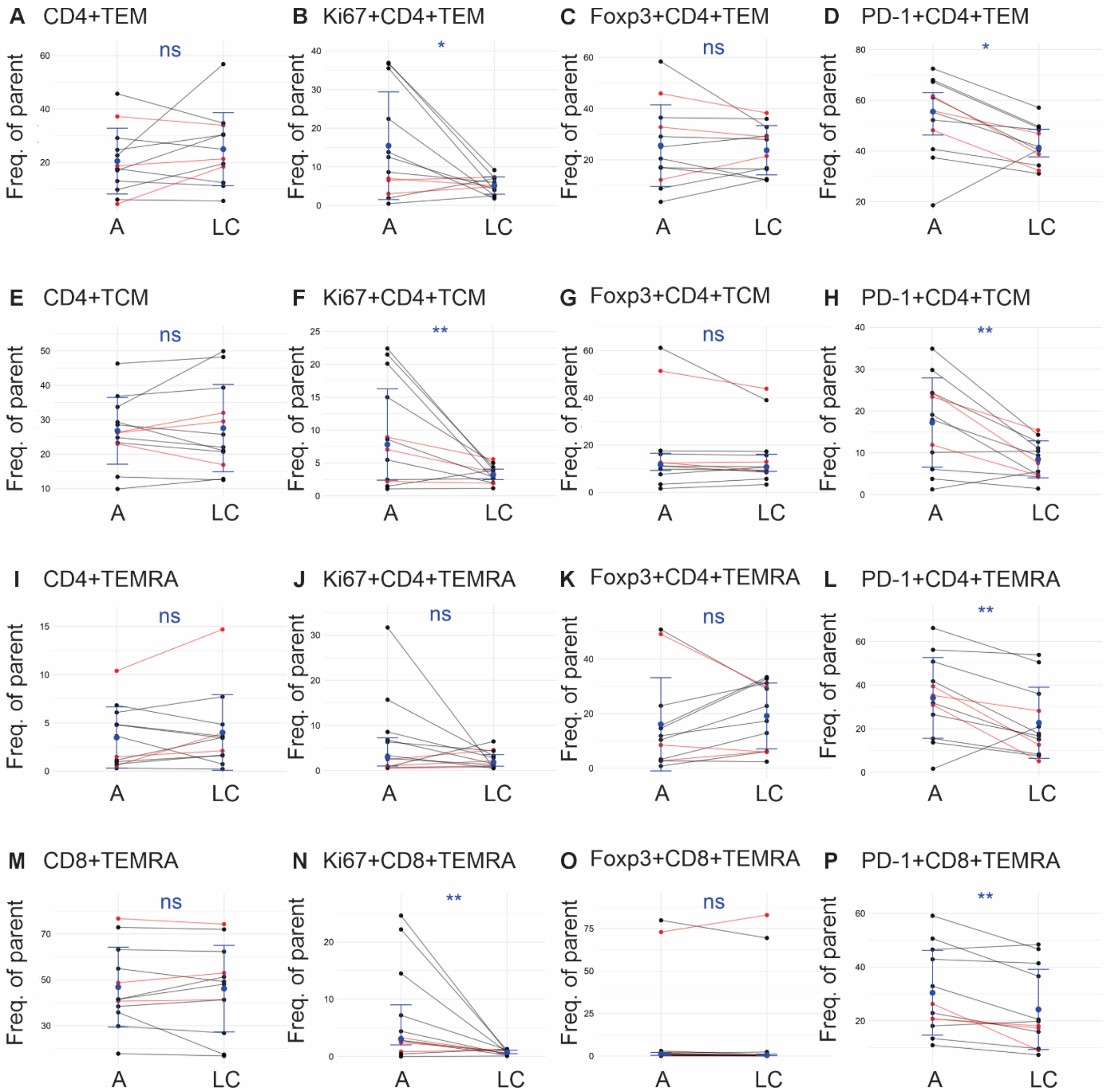

Supporting Information Figure 2. CD4+ and CD8+ memory T-cell subset frequencies and functional markers in paired samples (n=12) in acute and late convalescent (LC) stages. Panels show the frequency of effector memory T cells (CD45RA-CCR7-, TEMs, A), central memory T cells (CD45RA-CCR7+, TCMs, E), and terminal effector memory RA+ T cells

(CD45RA+CCR7-, TEMRAs, I for CD4+; M for CD8+) within their respective parent populations. For each subset, Ki67 (B, F, J, N), Foxp3 (C, G, K, O), and PD-1 (D, H, L, P) frequencies are displayed. Paired t-test or Wilcoxon signed-rank test with FDR correction were used with paired group comparison, depending on the data distribution. Patients aged over 65 years are indicated by red dots, and those under 65 years by black dots. Data are presented as mean ± SD (blue). **P*< 0.05, ***P*<0.01. ns=no statistical significance.

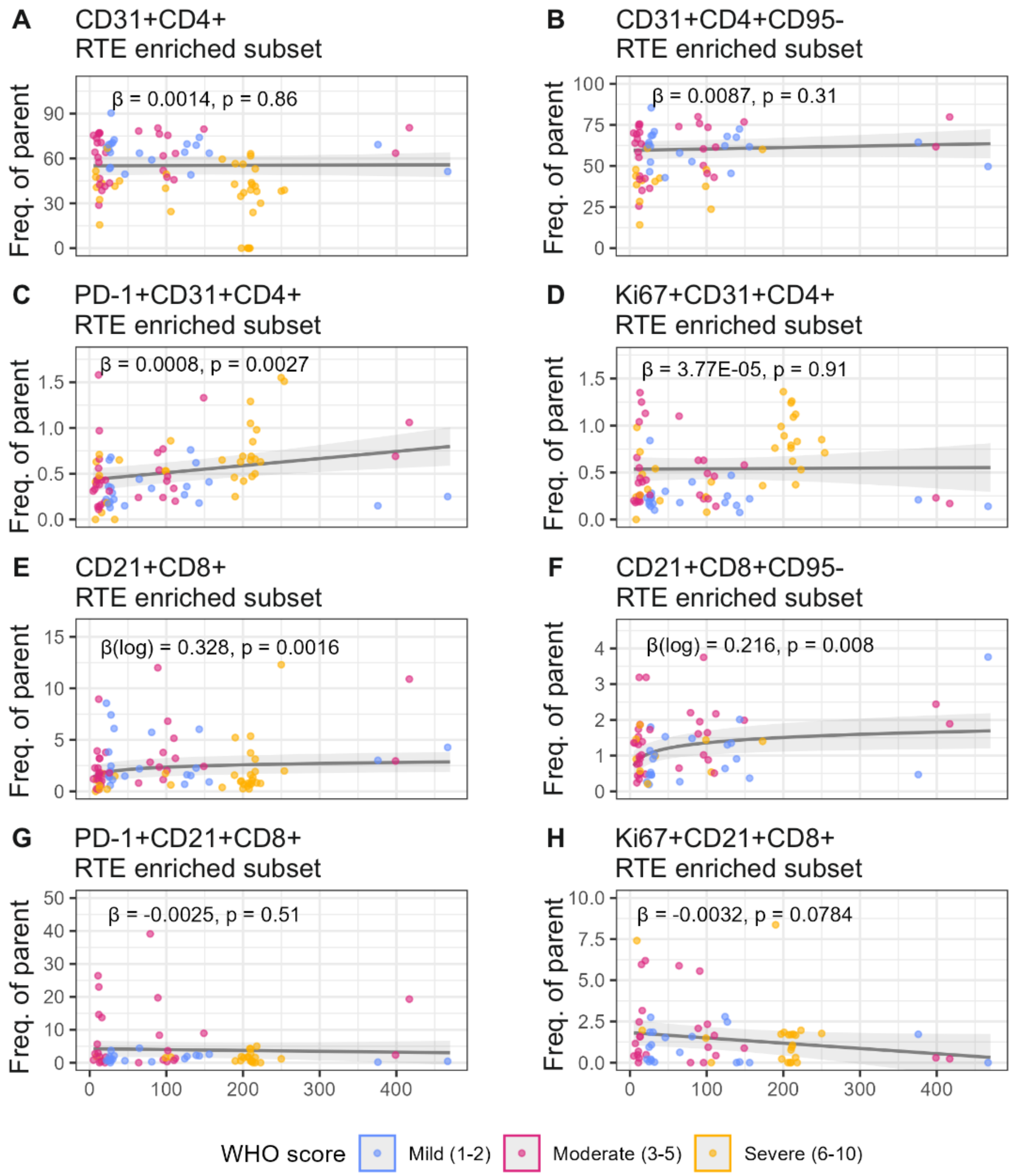

Supporting Information Figure 3. Restricted Maximum Likelihood (REML)-based linear mixed-effects modeling of longitudinal changes in cell populations across all 63 patients relative to days since symptom onset (dsso). The frequency of CD31+ CD4+ RTE-enriched subset of total (A) and CD95- (B) CD4+ naïve T cells. The frequency of PD-1+ (C) and Ki67+ (D) of CD31+ CD4+ RTE-enriched subset. The frequency of CD21+ CD8+ RTE-enriched subset of total (E) and CD95- (F) CD8+ naïve T cells. The frequency of PD-1+ (G) and Ki67+ (H) of CD21+ CD8+ RTE-enriched subset. WHO score is designated with different colors.

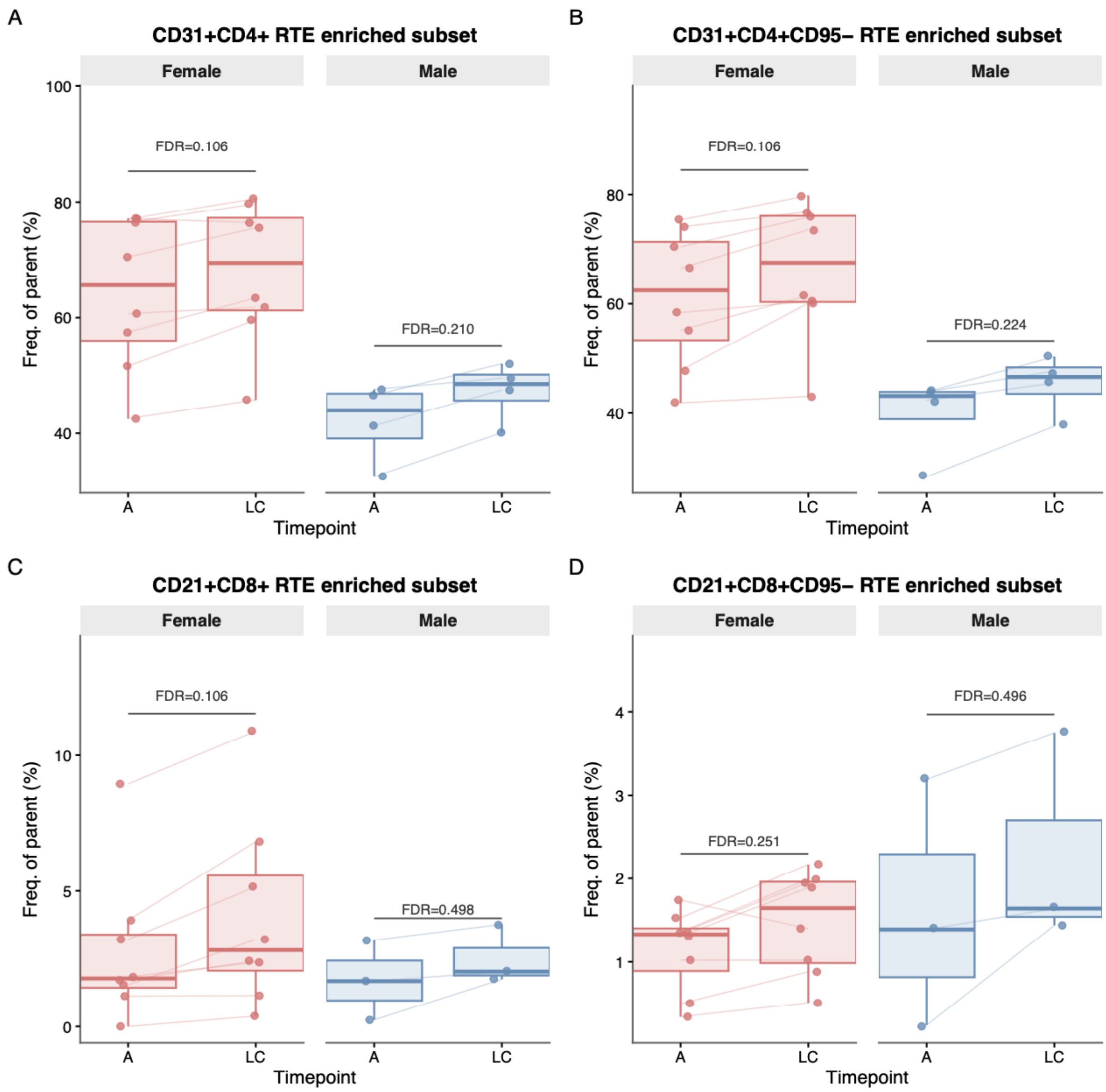

Supporting Information Figure 4. Sex-stratified analysis within paired samples (n=12) in acute and late convalescent (LC) stages. The frequency of CD31+ CD4+ (A) and CD31+ CD4+ CD95- (B) RTE-enriched subset of total and CD95- CD4+ naïve T cells respectively.

The frequency of CD21+ CD8+ (C) and CD21+ CD8+ CD95- (D) RTE-enriched subset of total and CD95- CD8+ naïve T cells respectively. Paired Wilcoxon signed-rank test with FDR correction was applied. Data shown as mean ± SD (blue).

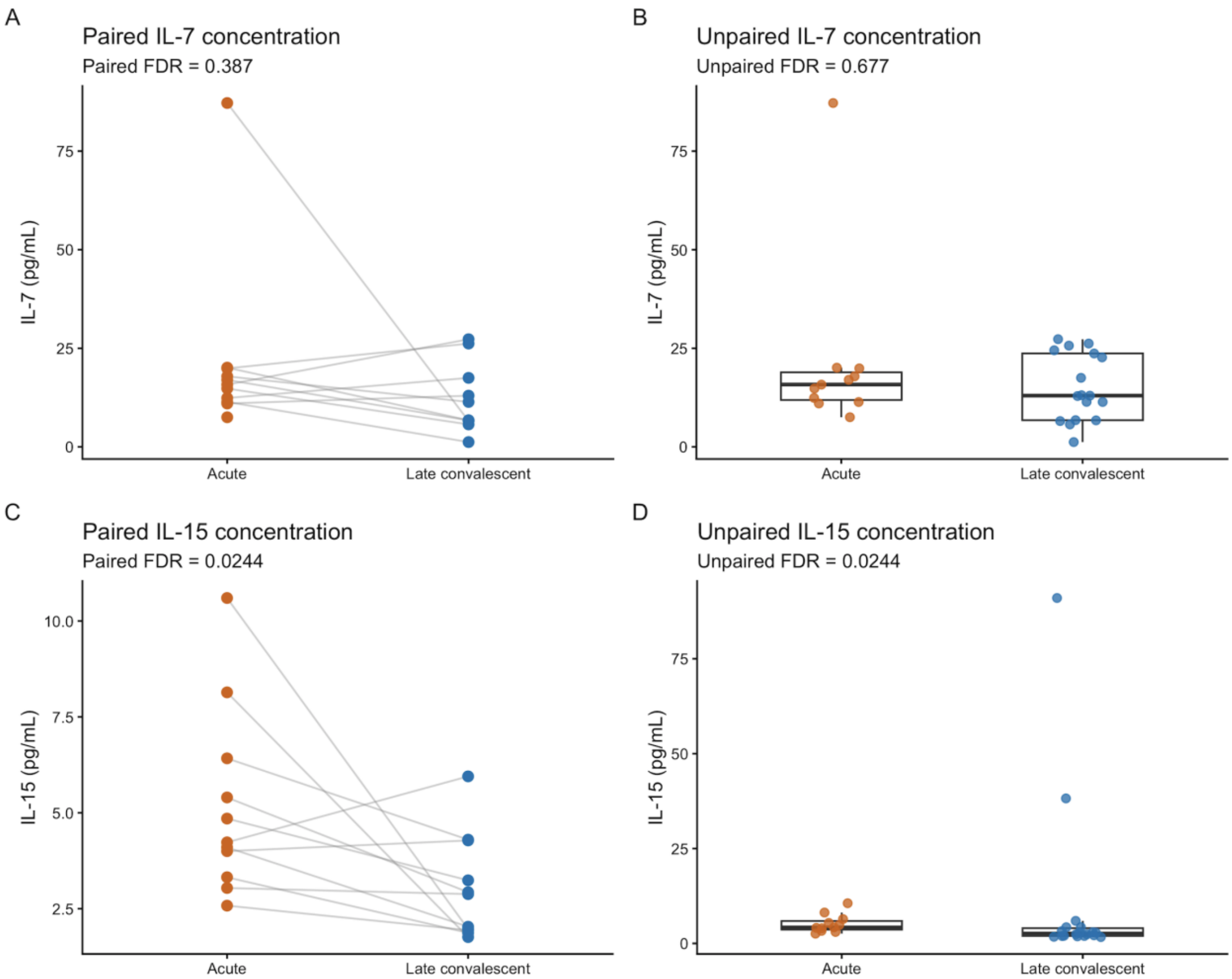

Supporting Information Figure 5. Plasma IL-7 and IL-15 concentrations in acute (n=11) and late convalescent (LC) (n=18) COVID-19 stages. IL-7 concentration in paired (A) and unpaired (B) acute and LC samples. IL-15 concentrations in paired (C) and unpaired (D) acute and LC samples. Paired t-test or Wilcoxon signed-rank test with FDR correction was applied. Data shown as mean ± SD.

Supporting Information Figure 6. Gating strategy for recent thymic emigrants

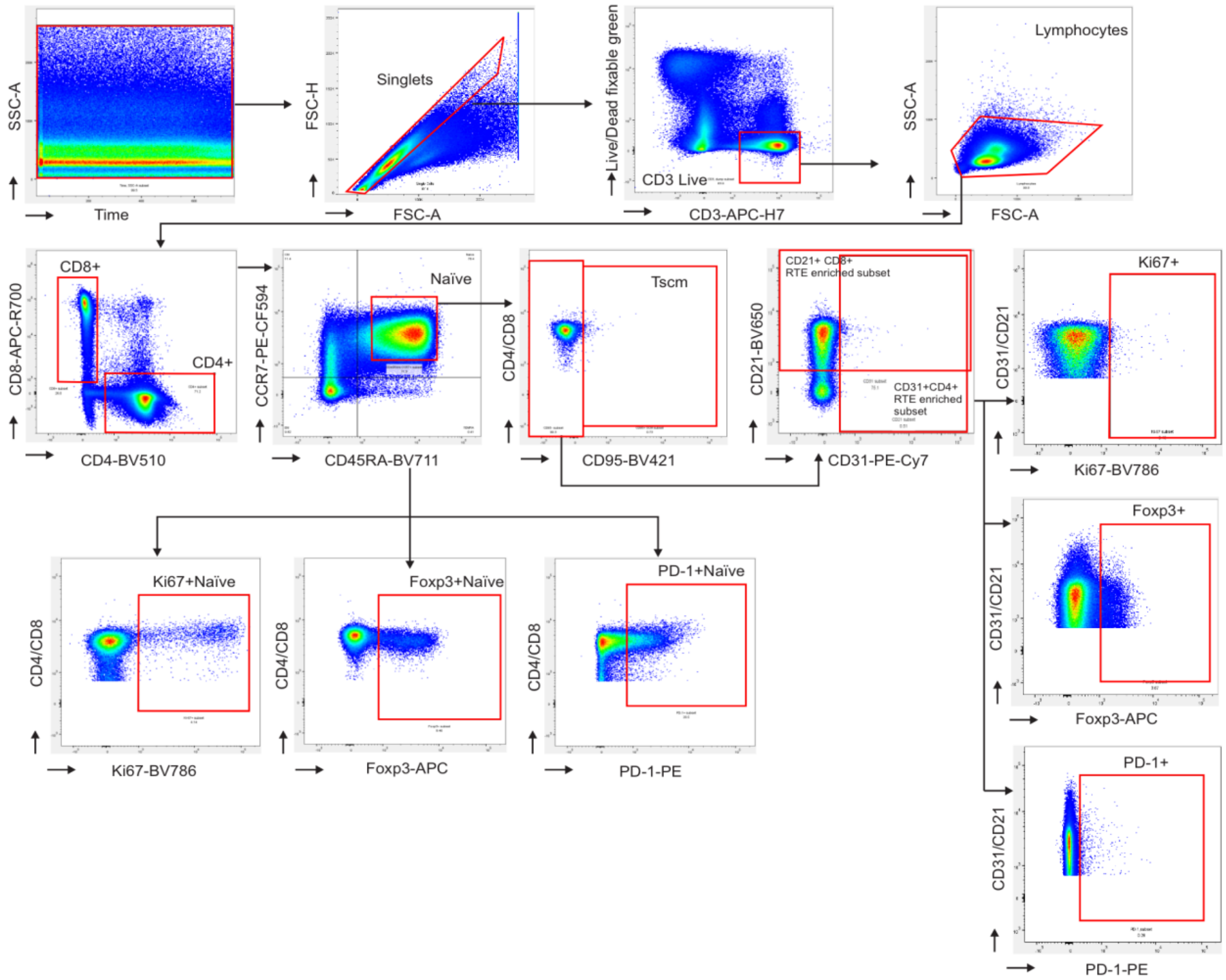

References for supplementary information

1. WHO Working Group on the Clinical Characterisation and Management of COVID-19 infection. A minimal common outcome measure set for COVID-19 clinical research. Lancet Infect Dis. 2020 Aug;20(8):e192–7.

2. Heikkilä N, Hetemäki I, Sormunen S, Isoniemi H, Kekäläinen E, Saramäki J, Arstila TP. Peripheral differentiation patterns of human T cells. European Journal of Immunology. 2022 Jun;52(6):882-894.

3. Bates D, Mächler M, Bolker B, Walker S. Fitting linear mixed-effects models Usinglme4. J Stat Softw [Internet]. 2015;67(1). Available from: http://dx.doi.org/10.18637/jss.v067.i01

4. Lüdecke D. sjPlot: Data Visualization for Statistics in Social Science [Internet]. CRAN: Contributed Packages. The R Foundation; 2013. Available from: http://dx.doi.org/10.32614/cran.package.sjplot
